# Inhibition of hepatocellular carcinoma growth by adenovirus-mediated expression of human telomerase reverse transcriptase COOH-27 terminal polypeptide in mice

**DOI:** 10.3892/ol.2013.1470

**Published:** 2013-07-16

**Authors:** LEI HE, HAN-XIAN GONG, XIANG-PEN LI, YI-DONG WANG, YI LI, JUN-JIAN HUANG, DAN XIE, HSIANG-FU KUNG, YING PENG

**Affiliations:** 1 Department of Neurology, Sun Yat-Sen Memorial Hospital, Sun Yat-Sen University, Guangzhou, Guangdong 510120, P.R. China; 2 Laboratory of Tumor and Molecular Biology, Beijing Institute of Biotechnology, Beijing 100071, P.R. China; 3 State Key Laboratory of Oncology in South China, Cancer Center, Sun Yat-Sen University, Guangzhou, Guangdong 510120, P.R. China; 4 Stanley Ho Center for Emerging Infectious Diseases, The Chinese University of Hong Kong, Shatin, Hong Kong, SAR, P.R. China

**Keywords:** hepatocellular carcinoma, cytotoxic T lymphocytes, gene therapy, immunotherapy, hTERTC27

## Abstract

A 27-kDa C-terminal fragment of human telomerase reverse transcriptase, hTERTC27, has previously been reported to inhibit the growth and tumorigenicity of HeLa human cervical cancer cells and U87-MG human glioblastoma multiforme cells. However, the antitumor effects of hTERTC27 in hepatoma and its underlying mechanisms are unclear. In the current study, the therapeutic effect of hTERTC27, mediated by recombinant adenovirus, in hepatocellular carcinoma (HCC) was explored *in vitro* and *in vivo* to investigate the possible mechanisms. The results indicated that recombinant adenovirus carrying hTERTC27 (rAdv-hTERTC27) effectively inhibited the growth and induced apoptosis of the Hepa 1–6 HCC cells. Dendritic cells transduced with rAdv-hTERTC27 were highly effective at inducing antigen-specific T cell proliferation and increasing the activated cytotoxicity of T cells against Hepa 1–6 cells. HCC was inhibited significantly when a single dose of 5×10^7^ pfu rAdv-hTERTC27 was administered intravenously. In summary, the results of this study demonstrated that rAdv-hTERTC27 may serve as a reagent for intravenous administration when combined with telomerase-based gene therapy and immunotherapy for cancer.

## Introduction

Hepatocellular carcinoma (HCC) is one of the most common types of malignancy worldwide, leading to >500,000 mortalities every year (1). Conventional chemotherapy and radiation treatments for HCC have been disappointing, with an overall 5-year survival rate of <10% (2). Although surgical resection has been considered to be the treatment methodology with the most curative potential, only an extremely small proportion of patients with primary liver cancer benefit transiently from surgical treatment, as recurrence rates are high following surgery. The majority of patients present with advanced-stage cancer and chronic hepatic dysfunction, limiting available surgery options (3,4). Other therapeutic approaches, including local alcohol injection, hepatic arterial immobilization and radiotherapy have not been found to significantly improve prognosis. These results highlight the urgent requirement for new therapies for HCC treatment. Gene therapy and immunotherapy are promising methods and extremely important. Gene therapy for malignant neoplasms has received considerable attention within the field and extensive experience associated with gene therapy, including toxicity, pharmacology and clinical indications, has been gained and reported (5,6).

Human telomerase reverse transcriptase (hTERT) has been identified as the catalytic enzyme required for telomere elongation. hTERT is expressed in the majority of tumor cells but is rarely expressed in human adult cells. It has been reported that 80–90% of HCCs express hTERT, and so the enzyme is a potential target in gene therapy for HCC (7,8). Adenovirus-mediated delivery of hTERT polypeptides into tumor cells is a well-studied approach that facilitates the eradication of tumors (9). hTERTC27, a 27-kDa C-terminal polypeptide of hTERT, is capable of inducing telomere dysfunction and anaphase chromosome end-to-end fusions in hTERT-positive HeLa cells. Overexpression of hTERTC27 also inhibits HeLa cell growth and tumorigenicity in nude mouse xenografts (10). Notably, the actions of hTERTC27 are mediated without perturbing the endogenous telomerase activity, thereby minimizing the potential side effects on telomerase-positive reproductive and proliferative cells of renewal tissues in antitelomerase therapies (11,12). In addition, the antitumor effect of hTERTC27 has been explored by delivering this gene to human glioblastoma multiforme cells using adeno-associated virus (AAV). It has been reported that intratumoral injection of recombinant AAV carrying hTERTC27 (rAAV-hTERTC27) is highly potent in inhibiting the growth of human U87-MG glioblastoma cells in athymic nude mice (13). In our previous study, it was demonstrated that hTERTC27 carried by adenovirus is able to augment the concentration of interleukin-2 (IL-2) and interferon-γ (IFN-γ) and induce antigen-specific cytotoxic T lymphocytes (CTLs) against glioma cells *in vitro,* indicating that adenovirus-delivered hTERTC27 may prolong survival time and inhibit the growth of glioma-bearing mice (14).

In the present study, hTERTC27 was delivered into murine HCC cells by adenovirus and its efficacy was observed to further explore the possible involvement of an immune response in cancer regression mediated by hTERTC27.

## Materials and methods

### Cell culture

Mouse HCC cells, Hepa 1–6, were obtained from the American Type Culture Collection (Rockville, MD, USA). The cell line was cultured in DMEM supplemented with 10% heat inactivated fetal bovine serum, 100 U/ml penicillin and 100 μg/ml streptomycin (Invitrogen Life Technologies, Carlsbad, CA, USA). The cells were incubated at 37°C in 5% CO_2_ and passaged every three days.

### Animals

C57BL/6 mice (5–8 weeks old) were purchased from Guangdong Medical Experimental Animal Center (Guangzhou, China) and were housed under aseptic conditions. All experimental protocols were performed in accordance with National Institutes of Health Guidelines and approved by the Animal Care and Use Committee of Sun Yat-sen University (Guangzhou, China).

### Cell proliferation assay

Hepa 1–6 cells were seeded at a density of 1.0×10^4^ cells/well in 96-well culture plates, transfected with rAdv-hTERTC27 or rAdv-EGFP at a multiplicity of infection of 30, and then incubated for 48 h. The presence of viable cells was tested through CCK-8 colorimetric assays (Dojindo Molecular Technologies, Inc., Gaithersburg, MD, USA) according to the manufacturer’s instructions. Absorbance values (at 450 nm), proportional to the number of living cells, were recorded for the culture medium of each sample using a Bio-Rad model 550 microplate reader (Hercules, CA, USA).

### Detection of apoptosis in vitro

Following 48 h of incubation and treatment as described, cells were stained with propidium iodide (PI) solution and Hochest 33258, as described previously (15,16) and observed with a fluorescence microscope (model IX70; Olympus, Tokyo, Japan). Cellular apoptosis was also detected using a Cell Death Detection ELISA Plus kit (Roche Diagnostics, Mannheim, Germany) according to the manufacturer’s instructions.

### rAdv-hTERTC27 transduction of dendritic cells (DCs) and induction of immune response

To determine the DC induction of CTL cytotoxicity against HCC cell lines, DCs and T cells were obtained as described previously (14). T cells were cocultured with DCs in a 24-well culture plate in 1 ml complete RPMI-1640 medium for 72 h. CTLs were collected and used as effector cells in CTL assays against Hepa 1–6 cells. The Hepa 1–6 cells, as target (T) cells, were placed in 96-well culture plates at 1×10^4^ cells/well and cocultured with effector (E) cells (CTL) at the indicated ratios (E:T = 5:1, 20:1 and 40:1) for 72 h. The cytotoxic activities were determined by CCK-8 colorimetric assays.

### HCC inoculation and intravenous injection of rAdv-hTERTC27

Hepa 1–6 cells were harvested during the exponential growth phase and washed twice in PBS. The cells were resuspended in PBS at a density of 5×10^7^ cells/ml and 0.1 ml (5×10^6^ cells/ml) of the cell suspension was injected directly into the hepatic capsule of the C57BL/6 mice. Mice were divided into four groups (eight mice/group) 7 days following tumor cell inoculation, and were treated under the following conditions: groups 1 and 2 received viral injections of 5.0×10^7^ pfu rAdv-hTERTC27 and an equal volume of the hTERTC27 polypeptide, respectively, representing treatment groups; groups 3 and 4 received 5.0×10^7^ pfu rAdv-EGFP and an equal volume of PBS, respectively, representing control groups. All treatments were administered via the tail vein. For the welfare of animals in experimental neoplasia, mice with tumor burdens >10% of their body weight were sacrificed immediately. Otherwise, 4 weeks following treatment, the mice were sacrificed under ether anesthesia and the total volume of the tumor (mm^3^) was calculated using the following formula: V = 1/2 × ab^2^, where a and b represent the long and short diameters of the tumor, respectively.

### Statistical analysis

Data are presented as the means ± SD. Statistical significance was assessed by one-way analysis of variance and Student’s t-test. Prism 5.0 (GraphPad Software, San Diego, CA, USA) was used for all calculations. P<0.05 was considered to indicate a statistically significant difference.

## Results

### Overexpression of hTERTC27 regulates Hepa 1–6 cell viability and apoptosis

**Figure 1:**
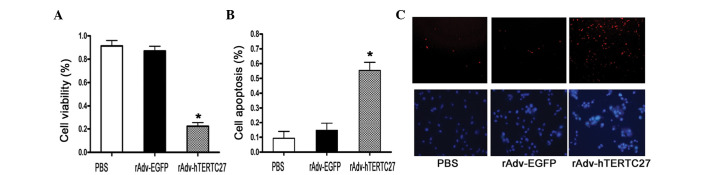
rAdv-hTERTC27 inhibits growth and induces apoptosis of Hepa 1–6 hepatocellular carcinoma cells. Hepa 1–6 cells were infected with recombinant adenovirus or vehicle for 48 h. (A) Cell viability was assessed using a CCK-8 assay and the results are presented as the mean ± SD of three independent experiments. ^*^P<0.05, vs. PBS and rAdv-EGFP. (B) Cell apoptosis was evaluated by a cell death detection kit and the results are presented as the mean ± SD of three independent experiments. ^*^P<0.05, vs. PBS and rAdv-EGFP. (C) Apoptotic cells were determined by PI and Hochest 33258 staining. The red and blue colors represent apoptotic cells (magnification, ×200). rAdv, recombinant adenovirus; hTERTC27, 27-kDa C-terminal fragment of human telomerase reverse transcriptase; PI, propidium iodide.

To test the effect of rAdv-hTERTC27 on Hepa 1–6 cells, cellular proliferation and apoptosis detection assays were performed. As demonstrated in Fig. 1, rAdv-hTERTC27 significantly inhibited the proliferation of the Hepa 1–6 cells (Fig. 1A). Cellular apoptosis was induced by rAdv-hTERTC27 and determined by PI and Hochest staining, as well as cell death ELISA detection (Fig. 1B and C). These results indicated that hTERTC27 may induce hepatocellular carcinoma cell apoptosis effectively *in vitro*.

### rAdv-hTERTC27-DCs induce T lymphocyte proliferation and prime CTLs against Hepa 1–6 HCC cells in vitro

**Figure 2:**
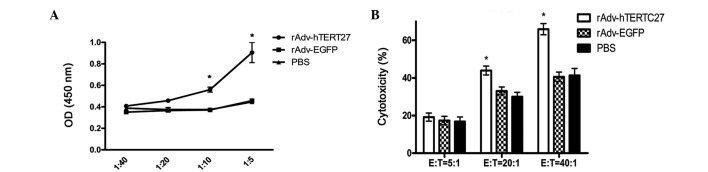
DCs transfected with rAdv-hTERTC27 induce T lymphocyte proliferation and prime cytotoxic activity of CTLs. (A) Following transfection with or without recombinant adenovirus for 24 h, DCs were cocultured with lymphocytes for 72 h at ratios of DC:T of 1:5, 1:10, 1:20 and 1:40. Following stimulation, the lymphocyte proliferation activity was analyzed using CCK-8 colorimetric assays. Experiments were repeated three times and representative results are presented. ^*^P<0.05, vs. PBS and rAdv-EGFP. (B) Allogeneic T cells were cocultured with rAdv-hTERTC27-DCs, rAdv-EGFP-DCs and PBS-DCs for 72 h. Hepa 1–6 cells, as target cells, were cocultured with effector cells (CTLs) at the indicated ratios (E:T = 5:1, 20:1 and 40:1) for 72 h. Cytotoxicity assay assessed by CCK-8 colorimetric assays. Experiments were repeated three times and results are presented. ^*^P<0.05, vs. PBS and rAdv-EGFP. DCs, dendritic cells; rAdv, recombinant adenovirus; hTERTC27, 27-kDa C-terminal fragment of human telomerase reverse transcriptase; CTL, cytotoxic T lymphocyte; E, effector; T, target.

rAdv-hTERTC27-DCs, rAdv-EGFP-DCs and PBS-DCs were cocultured with lymphocytes for 72 h at ratios of DC:T of 1:5, 1:10, 1:20 and 1:40. The results demonstrated that the effect of rAdv-hTERTC27-DC induction of T lymphocytes is markedly stronger than that by rAdv-EGFP-DCs and PBS-DCs when the DC:T ratio was ≥1:10 (P<0.05). However, no significant differences were identified between rAdv-EGFP and PBS (Fig. 2A). To test the hypothesis that the immune response is one of the predominant mechanisms responsible for the inhibition of tumor growth by rAdv-hTERTC27, mouse splenic T cells were further examined and primed *in vitro* with rAdv-hTERTC27-DCs to elicit cytotoxic reactivity against tumor cells. The results revealed that at E:T ratios of 5:1, 20:1 and 40:1, the lytic activity of CTLs was 16.16±2.75, 44.44±3.11 and 65.21±2.98%, respectively. However, the lytic activity of CTLs in rAdv-EGFP and PBS was 17.79±2.95, 33.65±3.16 and 40.54±3.18%, and 16.99±2.97, 30.57±2.64 and 48.72±3.45%, respectively. These results demonstrated that T cells primed with rAdv-hTERTC27-DCs *in vitro* were able to lyse tumor cells effectively as compared with the rAdv-EGFP-DCs and PBS-DCs, when the E:T ratio was ≥20:1 (P<0.05). However, no significant differences were identified between rAdv-EGFP and PBS (Fig. 2B).

### Intravenous injection of rAdv-hTERTC27 significantly inhibits the growth of HCC in mice

**Figure 3:**
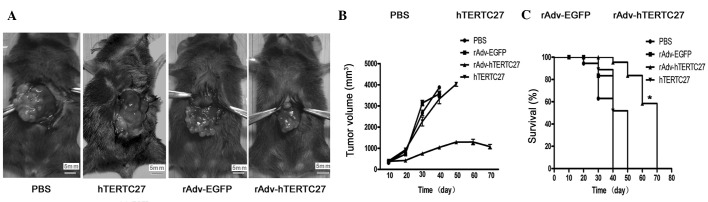
rAdv-hTERTC27 significantly reduces tumor size and prolongs survival rate of C57BL/6 mouse hepatic capsule injected with Hepa 1–6 cells. (A) Following 7-days post tumor cell injection, 5.0×10^7^ pfu of rAdv-hTERTC27 and rAdv-EGFP, and an equal volume of PBS and hTERTC27 polypeptide, were directly injected via the tail vein. Images of the mice were obtained following sacrifice. (B) rAdv-hTERTC27 markedly inhibits tumor volume. (C) rAdv-hTERTC27 significantly increases survival time. Mean life span of each mouse was observed daily. ^*^P<0.05 vs. PBS, rAdv-EGFP and hTERTC27.

To demonstrate the antitumor effect of hTERTC27 *in vivo*, an HCC model was established by implanting mouse Hepa 1–6 HCC cells into the hepatic capsule of C57BL/6 mice. PBS, rAdv-EGFP, rAdv-hTERTC27 and hTERTC27 polypeptides were injected 7 days later via the tail vein. As demonstrated in Fig. 3A, tumor volumes of rAdv-hTERTC27-treated mice were markedly smaller than those of rAdv-EGFP-, PBS- and hTERTC27-treated mice 4 weeks following injection. The mean tumor volume of the rAdv-hTERTC27, rAdv-EGFP, PBS and hTERTC27-treated mice was 1,012±51.63, 2,567±32.21, 2,789±29.12 and 2,412±34.51 mm^3^, respectively (Fig. 3B). In addition, the average life span of rAdv-EGFP-, PBS-and hTERTC27-treated mice bearing hepatocellular tumors were 34.5, 31 and 36 days, respectively. However, life span increased to 68 days in rAdv-hTERTC27-treated mice, although tumor-bearing mice died from progressive tumors. The prolonged survival rate of tumor-bearing mice was observed in the majority of rAdv-hTERTC27 groups compared with in rAdv-EGFP, PBS and hTERTC27 groups (Fig. 3C; P<0.05). The results demonstrated that the anti-tumor effects of rAdv-hTERTC27 are achieved effectively *in vivo*.

## Discussion

In the current study, the therapeutic effect of rAdv-hTERTC27 on HCC, *in vivo* and *in vitro,* was demonstrated. Results indicated that rAdv-hTERTC27 produces a longer-lasting effect on the generation of effective antitumor immunity as a reagent via intravenous administration.

The gene delivery vector of choice was the adenovirus, as it has numerous advantages, including a high gene transduction efficiency, stability in the bloodstream and an acceptable safety profile (17,18,19). However, systemic administration of conventional adenovirus is capable of leading to the acute accumulation of virus particles and transgene expression in the liver, which may cause severe hepatotoxicity. Therefore, the clinical application of the adenovirus for systemic administration has been limited. To determine the application of the adenovirus in systemic cancer gene therapy in clinical patients, the accumulation must be enhanced in target tumors and hepatic distribution must be minimized. As a universal tumor-associated antigen, hTERT is an ideal target for cancer therapy. It is well-known that hTERT is expressed by the majority of human forms of cancer but rarely in normal cells (20). The widespread expression of telomerase in tumors indicates that peptide fragments of hTERT may serve as tumor-specific antigens and this hypothesis has been confirmed in several studies (21,22). Therefore, in the present study, rAdv-hTERTC27 was developed as a TERT-targeting gene therapy.

Previously, it was demonstrated that overexpression of hTERTC27 inhibits HeLa cell growth and tumorigenicity in nude mouse xenografts (10). The antitumor actions of hTERTC27 are likely to be effected by increased apoptosis, necrosis and infiltration of polymorphonuclear leukocytes, reducing angiogenesis in glioblastoma (13). The results of the present study demonstrated that rAdv-hTERTC27 effectively reduces growth and increases apoptosis of Hepa 1–6 HCC cells *in vitro.* It also inhibited the tumor volume in HCC mouse models intravenously injected with rAdv-hTERTC27. The indicated statistically significant difference between the results for rAdv-hTERTC27 and hTERTC27 polypeptides may be accounted for by two hypotheses: The high efficiency of recombinant adenovirus as a gene delivery vector or, more importantly, rAdv-hTERTC27 may promote specific mouse mechanisms in the bloodstream that markedly augment the antitumor ability of hTERTC27.

In our previous study, the potential mechanisms underlying the effects of rAdv-hTERTC27 were explored and rAdv-hTERTC27 was demonstrated to increase T cell proliferation and augment the concentration of IL-2 and IFN-γ in the supernatant of T cells. In the current study, DCs infected with rAdv-hTERTC27 markedly increased the activated cytotoxicity of T cells against Hepa 1–6 cells. It is well-known that IFN-γ and IL-2 are critical for T cell responses. IFN-γ is primarily responsible for activating and regulating the development and persistence of CTLs (23,24). IL-2 is capable of inducing a distinct CTL effector function (25) and the administration of adenovirus vector-encoding mouse IL-2 (AdmIL-2) may augment the antitumor effect of TRAIL on tumors in mice (26). Activated T cells are able to produce IFN-γ and induce cytolysis of autologous tumor or semi-allogeneic targets by an MHC class I-restricted mechanism (27). DCs transduced with the recombinant adenovirus-encoding peptide may effectively induce antigen-specific T cell proliferation, augment the number of IFN-γ-secreting T-cells and induce antigen-specific CTLs capable of lysing target cells pulsed with the peptide (28,29). The immunoregulatory function of DCs induced by rAdv-hTERTC27 is capable of potentiating the initiation, expansion and effect or phases of an evolving adaptive T cell-mediated immune response that ultimately leads to the inhibition of tumor cells. By contrast, increased IFN-γ secretion by T cells may enhance the activation of CTLs again. Therefore, hTERTC27 gene-modified DCs are capable of generating a specific CTL response against various hTERT-expressing cancer cell lines. The susceptibility of tumor cell lines of various origins to lysis by telomerase-specific CTLs indicates that hTERTC27 may be used alone or in combination with other tumor antigens in immunotherapy against a wide range of cancer types. Adoptive immunotherapy with expanded antigen-specific CTLs may be an effective approach to treat cancer. However, the efficacy of the adenovirus to generate hTERTC27-specific CTLs indicates that this form of vaccination must also be considered in cases where potential adenovirus toxicity or high levels of neutralizing antibodies is a concern. In addition, DC-hTERTC27-based cancer vaccines that have the potential to maximize the protection against various hTERT^+^ tumor cells must be investigated, and the dose of recombinant adenovirus ought to be minimized to decrease the side effects of gene therapy and immunotherapy.

In the current study, an original HCC model in C57BL/6 mice was established. C57BL/6 mice were used in this model as Hepa 1–6 cell lines are derived from a chemically induced hepatoma from the C57BL/6 mouse (30). In addition, immunocompetent animal models are suitable for the assessment of immune responses elicited by hTERTC27. Tumor growth was inhibited significantly, compared with the control groups, when mice were administered with intravenous injection of a single dose of 5×10^7^ pfu rAdv-hTERTC27. Through the immunoregulatory function added to gene regulation, the dose of rAdv-hTERTC27 was decreased to a higher degree than was previously reported (31). Thus, the potential side effects of the adenovirus were lower, which was a concern with regards to gene therapy and immunotherapy.

In conclusion, the results of the current study demonstrate that rAdv-hTERTC27 induces tumor cell apoptosis in murine HCC models. We hypothesize that recombinant adenoviral constructs containing the hTERTC27 polypeptide represent promising intravenous drugs for use in clinical practice against HCC.
